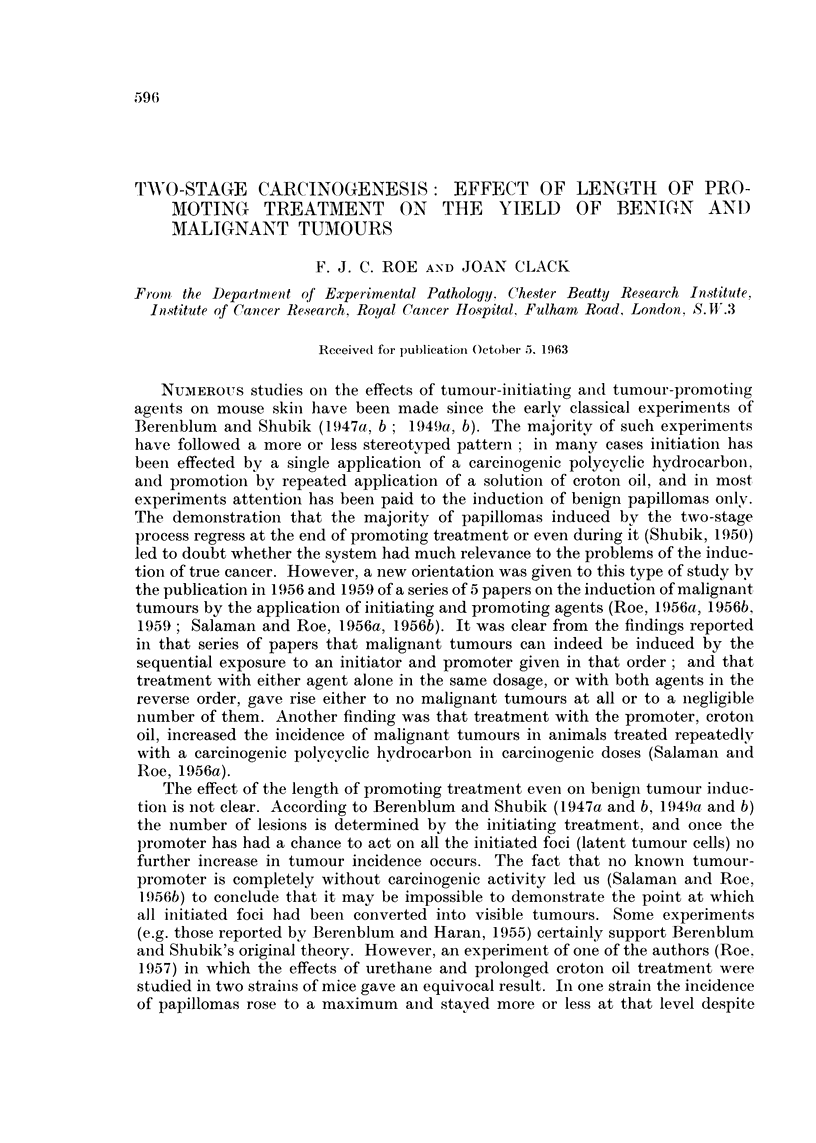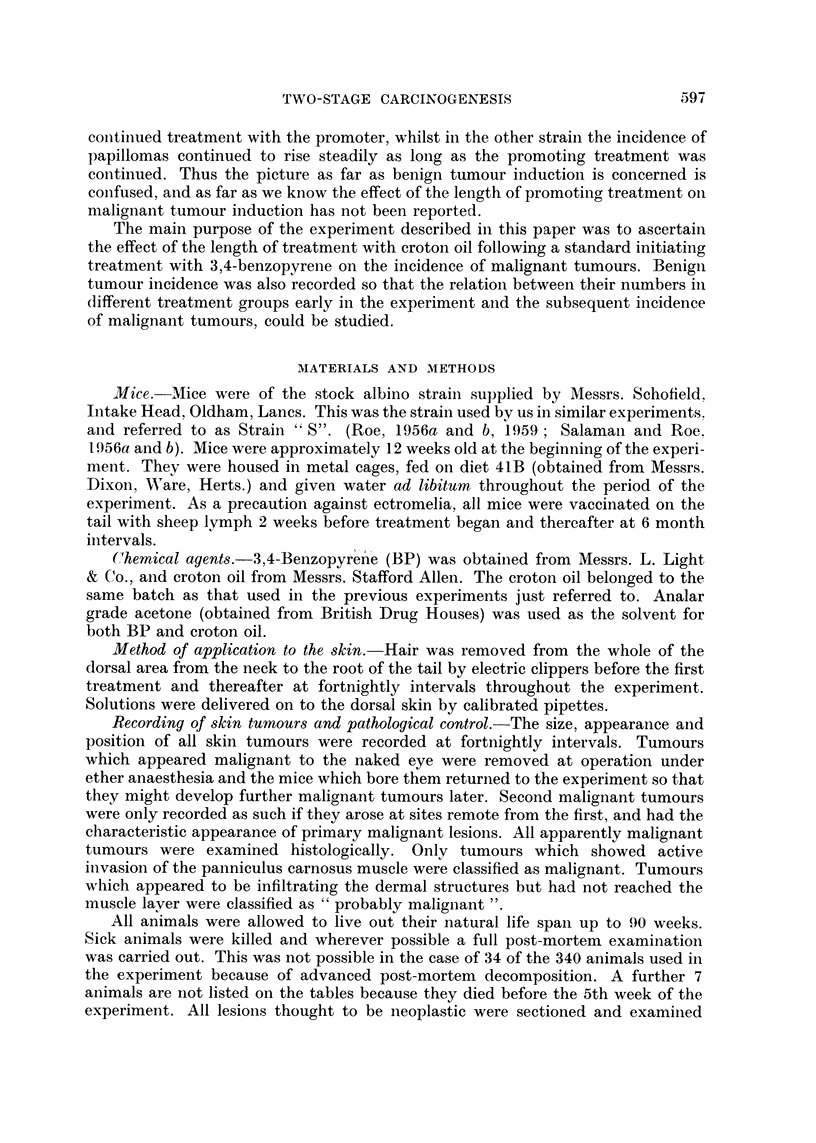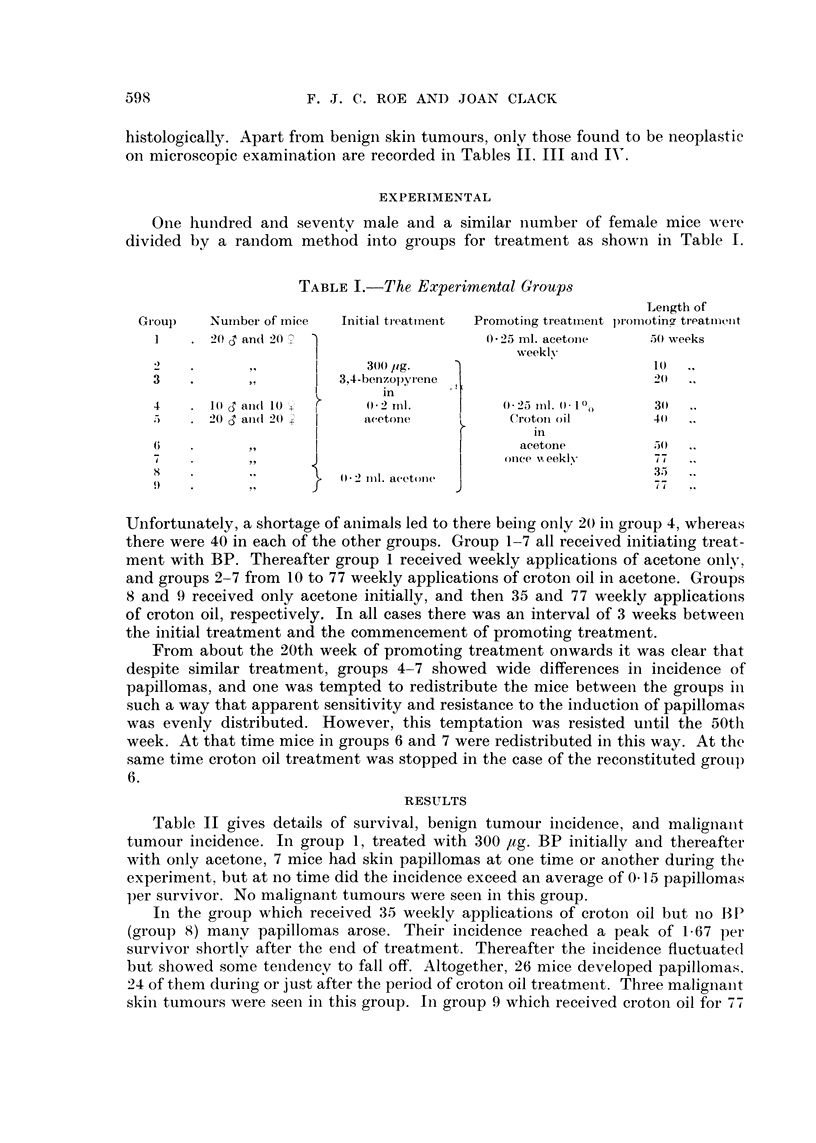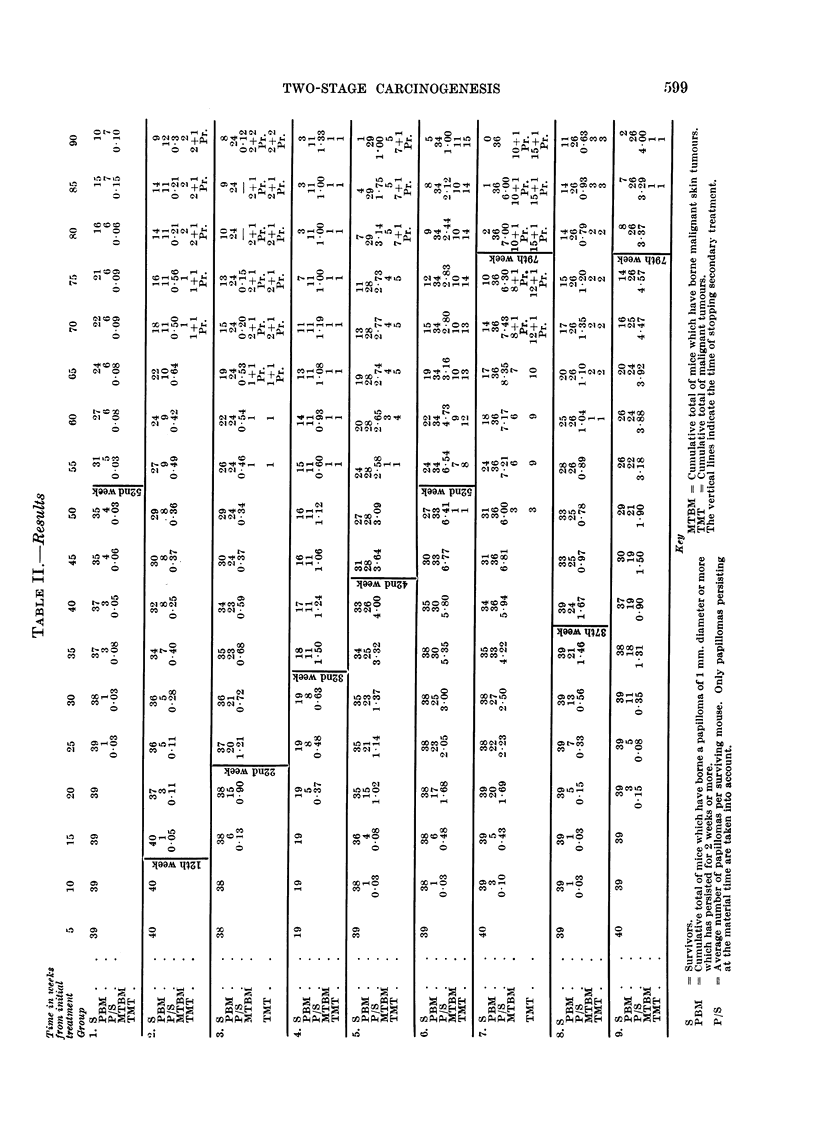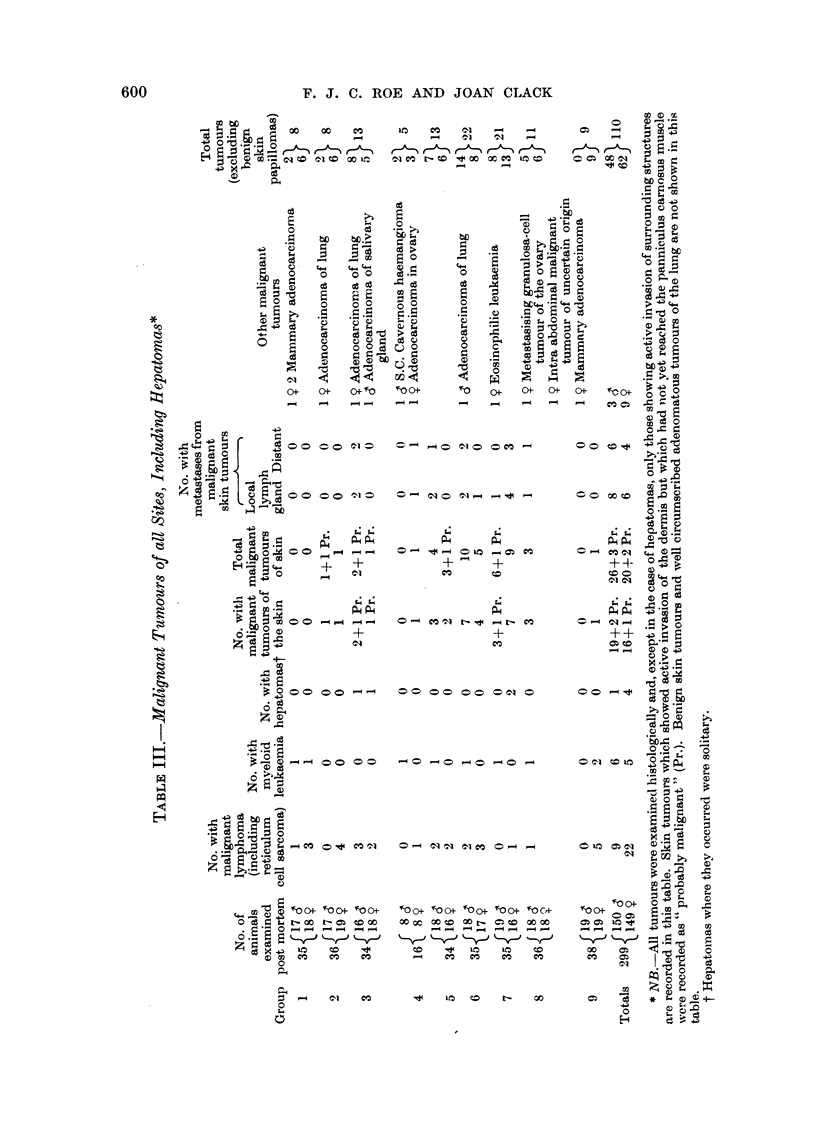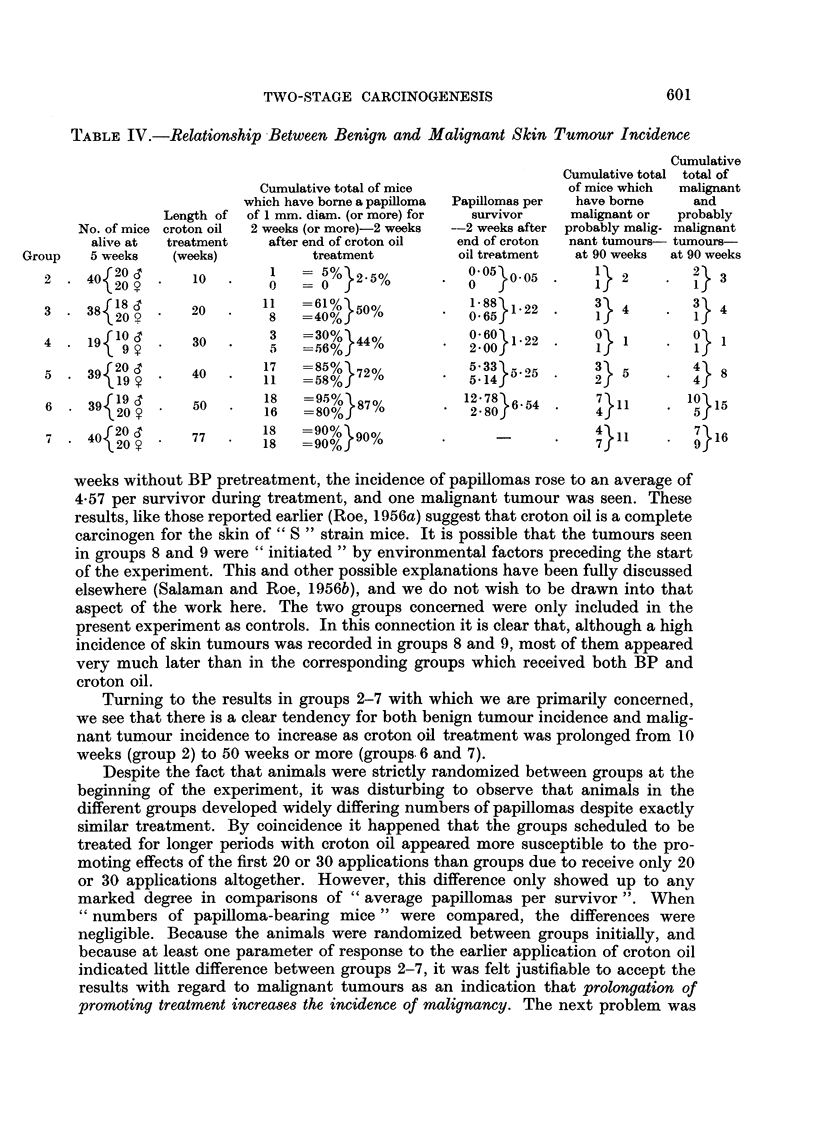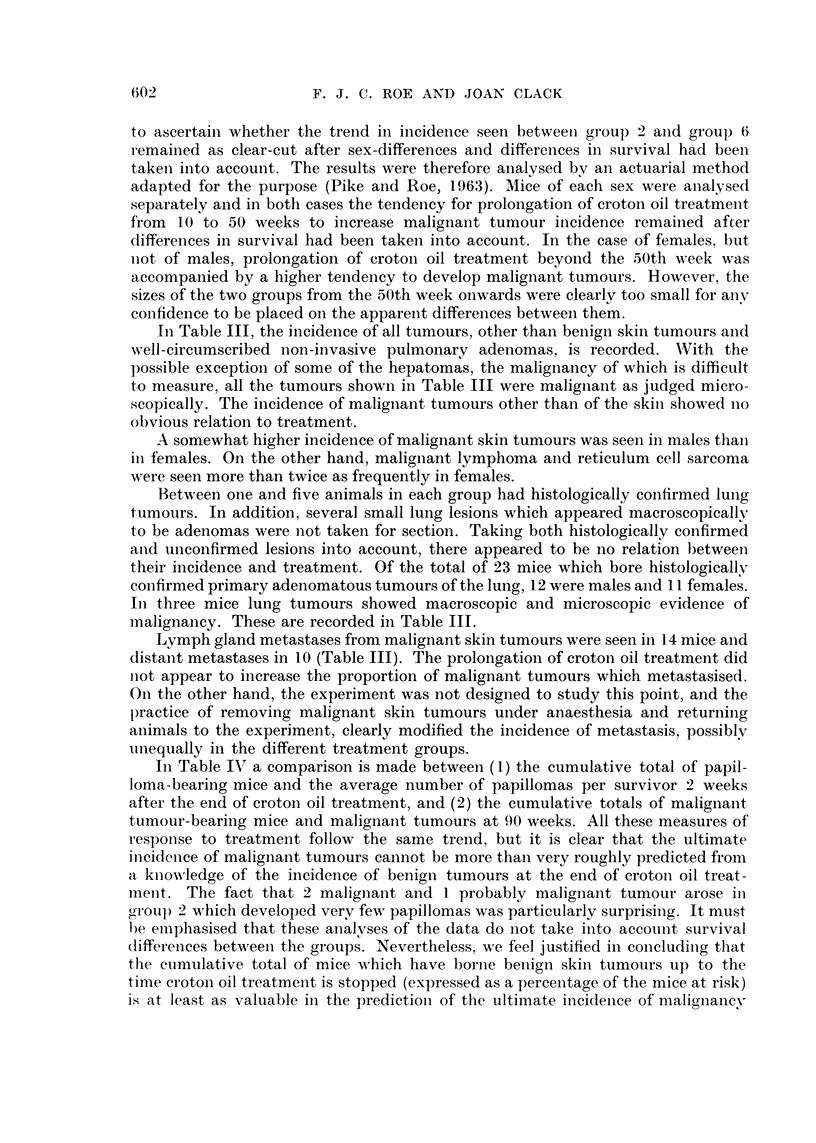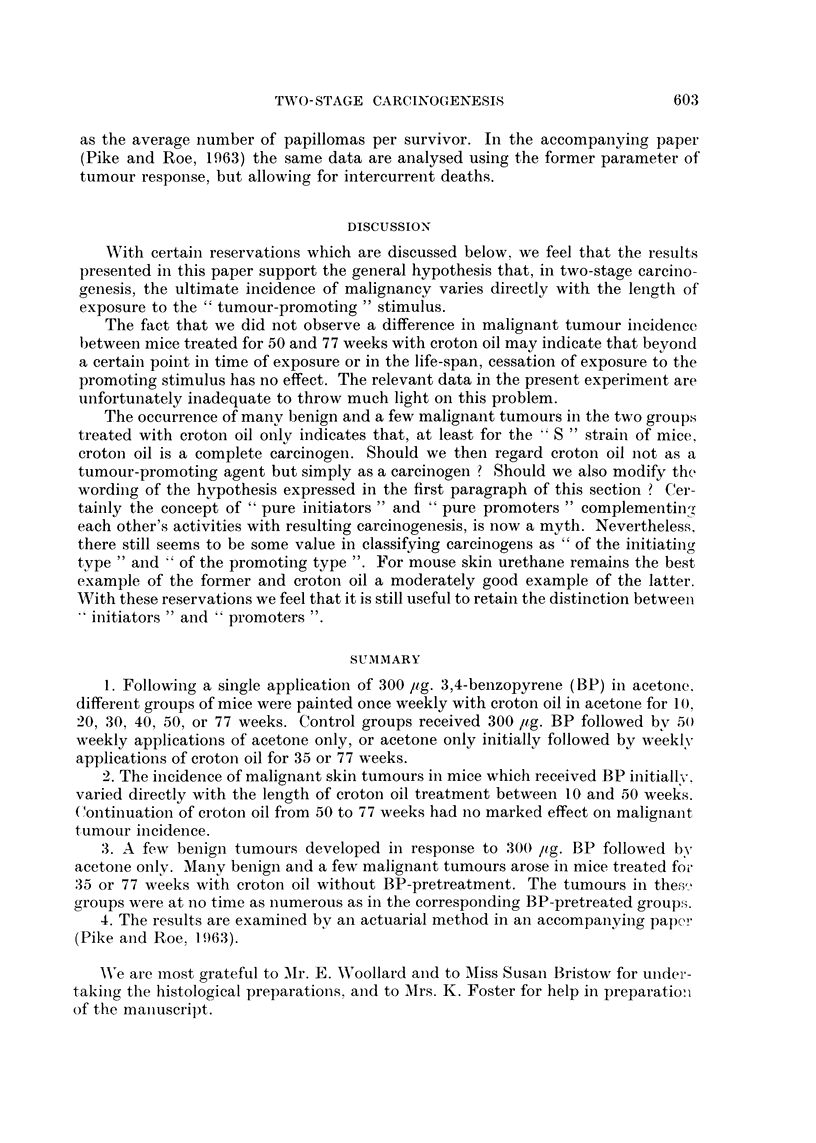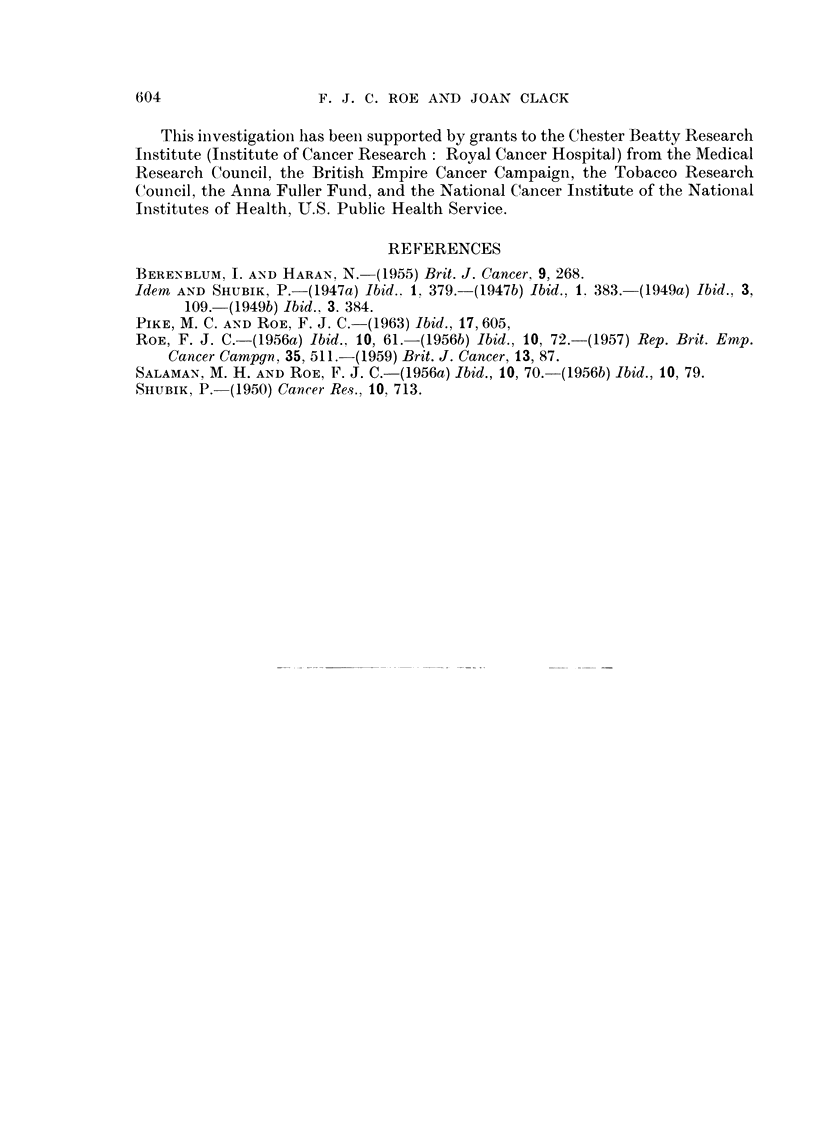# Two-Stage Carcinogenesis: Effect of length of Promoting Treatment on the Yield of Benign and Malignant Tumours

**DOI:** 10.1038/bjc.1963.79

**Published:** 1963-12

**Authors:** F. J. C. Roe, Joan Clack


### TWO-STAGE CARCINOGENESIS: EFFECT OF LENGTH OF PROMOTING TREATMENT ON THE YIELD OF BENIGN AND MALIGNANT TUMOURS

F. J. C. ROE AND JOAN CLACK

*From the Department of Experimental Pathology, Chester Beatty Research Institute, Institute of Cancer Research, Royal Cancer Hospital, Fulham Road, London, S.W.3*

Received for publication October 5, 1963

NUMEROUS studies on the effects of tumour-initiating and tumour-promoting agents on mouse skin have been made since the early classical experiments of Berenblum and Shubik (1947*a*, *b*; 1949*a*, *b*). The majority of such experiments have followed a more or less stereotyped pattern; in many cases initiation has been effected by a single application of a carcinogenic polycyclic hydrocarbon, and promotion by repeated application of a solution of croton oil, and in most experiments attention has been paid to the induction of benign papillomas only. The demonstration that the majority of papillomas induced by the two-stage process regress at the end of promoting treatment or even during it (Shubik, 1950) led to doubt whether the system had much relevance to the problems of the induction of true cancer. However, a new orientation was given to this type of study by the publication in 1956 and 1959 of a series of 5 papers on the induction of malignant tumours by the application of initiating and promoting agents (Roe, 1956*a*, 1956*b*, 1959; Salaman and Roe, 1956*a*, 1956*b*). It was clear from the findings reported in that series of papers that malignant tumours can indeed be induced by the sequential exposure to an initiator and promoter given in that order; and that treatment with either agent alone in the same dosage, or with both agents in the reverse order, gave rise either to no malignant tumours at all or to a negligible number of them. Another finding was that treatment with the promoter, croton oil, increased the incidence of malignant tumours in animals treated repeatedly with a carcinogenic polycyclic hydrocarbon in carcinogenic doses (Salaman and Roe, 1956*a*).

The effect of the length of promoting treatment even on benign tumour induction is not clear. According to Berenblum and Shubik (1947*a* and *b*, 1949*a* and *b*) the number of lesions is determined by the initiating treatment, and once the promoter has had a chance to act on all the initiated foci (latent tumour cells) no further increase in tumour incidence occurs. The fact that no known tumour-promoter is completely without carcinogenic activity led us (Salaman and Roe, 1956*b*) to conclude that it may be impossible to demonstrate the point at which all initiated foci had been converted into visible tumours. Some experiments (e.g. those reported by Berenblum and Haran, 1955) certainly support Berenblum and Shubik's original theory. However, an experiment of one of the authors (Roe, 1957) in which the effects of urethane and prolonged croton oil treatment were studied in two strains of mice gave an equivocal result. In one strain the incidence of papillomas rose to a maximum and stayed more or less at that level despite continued treatment with the promoter, whilst in the other strain the incidence of papillomas continued to rise steadily as long as the promoting treatment was continued. Thus the picture as far as benign tumour induction is concerned is confused, and as far as we know the effect of the length of promoting treatment on malignant tumour induction has not been reported.

The main purpose of the experiment described in this paper was to ascertain the effect of the length of treatment with croton oil following a standard initiating treatment with 3,4-benzopyrene on the incidence of malignant tumours. Benign tumour incidence was also recorded so that the relation between their numbers in different treatment groups early in the experiment and the subsequent incidence of malignant tumours, could be studied.

### MATERIALS AND METHODS

*Mice.*—Mice were of the stock albino strain supplied by Messrs. Schofield, Intake Head, Oldham, Lancs. This was the strain used by us in similar experiments, and referred to as Strain "S". (Roe, 1956*a* and *b*, 1959; Salaman and Roe, 1956*a* and *b*). Mice were approximately 12 weeks old at the beginning of the experiment. They were housed in metal cages, fed on diet 41B (obtained from Messrs. Dixon, Ware, Herts.) and given water *ad libitum* throughout the period of the experiment. As a precaution against ectromelia, all mice were vaccinated on the tail with sheep lymph 2 weeks before treatment began and thereafter at 6 month intervals.

*Chemical agents.*—3,4-Benzopyrene (BP) was obtained from Messrs. L. Light & Co., and croton oil from Messrs. Stafford Allen. The croton oil belonged to the same batch as that used in the previous experiments just referred to. Analar grade acetone (obtained from British Drug Houses) was used as the solvent for both BP and croton oil.

*Method of application to the skin.*—Hair was removed from the whole of the dorsal area from the neck to the root of the tail by electric clippers before the first treatment and thereafter at fortnightly intervals throughout the experiment. Solutions were delivered on to the dorsal skin by calibrated pipettes.

*Recording of skin tumours and pathological control.*—The size, appearance and position of all skin tumours were recorded at fortnightly intervals. Tumours which appeared malignant to the naked eye were removed at operation under ether anaesthesia and the mice which bore them returned to the experiment so that they might develop further malignant tumours later. Second malignant tumours were only recorded as such if they arose at sites remote from the first, and had the characteristic appearance of primary malignant lesions. All apparently malignant tumours were examined histologically. Only tumours which showed active invasion of the panniculus carnosus muscle were classified as malignant. Tumours which appeared to be infiltrating the dermal structures but had not reached the muscle layer were classified as "probably malignant".

All animals were allowed to live out their natural life span up to 90 weeks. Sick animals were killed and wherever possible a full post-mortem examination was carried out. This was not possible in the case of 34 of the 340 animals used in the experiment because of advanced post-mortem decomposition. A further 7 animals are not listed on the tables because they died before the 5th week of the experiment. All lesions thought to be neoplastic were sectioned and examined histologically. Apart from benign skin tumours, only those found to be neoplastic on microscopic examination are recorded in Tables II, III and IV.

### EXPERIMENTAL

One hundred and seventy male and a similar number of female mice were divided by a random method into groups for treatment as shown in Table I.

TABLE I.—*The Experimental Groups*

| Group | Number of mice | Initial treatment | Promoting treatment | Length of promoting treatment |
|---|---|---|---|---|
| 1 | . 20 ♂ and 20 ♀ | | 0·25 ml. acetone weekly | 50 weeks |
| 2 | . ,, | 300 $\mu$g. | | 10 ,, |
| 3 | . ,, | 3,4-benzopyrene in | | 20 ,, |
| 4 | . 10 ♂ and 10 ♀ | 0·2 ml. | 0·25 ml. 0·1% | 30 ,, |
| 5 | . 20 ♂ and 20 ♀ | acetone | Croton oil in | 40 ,, |
| 6 | . ,, | | acetone | 50 ,, |
| 7 | . ,, | | once weekly | 77 ,, |
| 8 | . ,, | 0·2 ml. acetone | | 35 ,, |
| 9 | . ,, | | | 77 ,, |

Unfortunately, a shortage of animals led to there being only 20 in group 4, whereas there were 40 in each of the other groups. Group 1–7 all received initiating treatment with BP. Thereafter group 1 received weekly applications of acetone only, and groups 2–7 from 10 to 77 weekly applications of croton oil in acetone. Groups 8 and 9 received only acetone initially, and then 35 and 77 weekly applications of croton oil, respectively. In all cases there was an interval of 3 weeks between the initial treatment and the commencement of promoting treatment.

From about the 20th week of promoting treatment onwards it was clear that despite similar treatment, groups 4–7 showed wide differences in incidence of papillomas, and one was tempted to redistribute the mice between the groups in such a way that apparent sensitivity and resistance to the induction of papillomas was evenly distributed. However, this temptation was resisted until the 50th week. At that time mice in groups 6 and 7 were redistributed in this way. At the same time croton oil treatment was stopped in the case of the reconstituted group 6.

### RESULTS

Table II gives details of survival, benign tumour incidence, and malignant tumour incidence. In group 1, treated with 300 $\mu$g. BP initially and thereafter with only acetone, 7 mice had skin papillomas at one time or another during the experiment, but at no time did the incidence exceed an average of 0·15 papillomas per survivor. No malignant tumours were seen in this group.

In the group which received 35 weekly applications of croton oil but no BP (group 8) many papillomas arose. Their incidence reached a peak of 1·67 per survivor shortly after the end of treatment. Thereafter the incidence fluctuated but showed some tendency to fall off. Altogether, 26 mice developed papillomas, 24 of them during or just after the period of croton oil treatment. Three malignant skin tumours were seen in this group. In group 9 which received croton oil for 77

TABLE IV.—*Relationship Between Benign and Malignant Skin Tumour Incidence*

| Group | No. of mice alive at 5 weeks | Length of croton oil treatment (weeks) | Cumulative total of mice which have borne a papilloma of 1 mm. diam. (or more) for 2 weeks (or more)—2 weeks after end of croton oil treatment | Papillomas per survivor—2 weeks after end of croton oil treatment | Cumulative total of mice which have borne malignant or probably malignant tumours—at 90 weeks | Cumulative total of malignant and probably malignant tumours—at 90 weeks |
|---|---|---|---|---|---|---|
| 2 | 40 {20 ♂<br>20 ♀ | 10 | 1 = 5%<br>0 = 0 } 2·5% | 0·05<br>0 } 0·05 | 1<br>1 } 2 | 2<br>1 } 3 |
| 3 | 38 {18 ♂<br>20 ♀ | 20 | 11 =61%<br>8 =40% } 50% | 1·88<br>0·65 } 1·22 | 3<br>1 } 4 | 3<br>1 } 4 |
| 4 | 19 {10 ♂<br>9 ♀ | 30 | 3 =30%<br>5 =56% } 44% | 0·60<br>2·00 } 1·22 | 0<br>1 } 1 | 0<br>1 } 1 |
| 5 | 39 {20 ♂<br>19 ♀ | 40 | 17 =85%<br>11 =58% } 72% | 5·33<br>5·14 } 5·25 | 3<br>2 } 5 | 4<br>4 } 8 |
| 6 | 39 {19 ♂<br>20 ♀ | 50 | 18 =95%<br>16 =80% } 87% | 12·78<br>2·80 } 6·54 | 7<br>4 } 11 | 10<br>5 } 15 |
| 7 | 40 {20 ♂<br>20 ♀ | 77 | 18 =90%<br>18 =90% } 90% | — | 4<br>7 } 11 | 7<br>9 } 16 |

weeks without BP pretreatment, the incidence of papillomas rose to an average of 4·57 per survivor during treatment, and one malignant tumour was seen. These results, like those reported earlier (Roe, 1956*a*) suggest that croton oil is a complete carcinogen for the skin of " S " strain mice. It is possible that the tumours seen in groups 8 and 9 were " initiated " by environmental factors preceding the start of the experiment. This and other possible explanations have been fully discussed elsewhere (Salaman and Roe, 1956*b*), and we do not wish to be drawn into that aspect of the work here. The two groups concerned were only included in the present experiment as controls. In this connection it is clear that, although a high incidence of skin tumours was recorded in groups 8 and 9, most of them appeared very much later than in the corresponding groups which received both BP and croton oil.

Turning to the results in groups 2–7 with which we are primarily concerned, we see that there is a clear tendency for both benign tumour incidence and malignant tumour incidence to increase as croton oil treatment was prolonged from 10 weeks (group 2) to 50 weeks or more (groups 6 and 7).

Despite the fact that animals were strictly randomized between groups at the beginning of the experiment, it was disturbing to observe that animals in the different groups developed widely differing numbers of papillomas despite exactly similar treatment. By coincidence it happened that the groups scheduled to be treated for longer periods with croton oil appeared more susceptible to the promoting effects of the first 20 or 30 applications than groups due to receive only 20 or 30 applications altogether. However, this difference only showed up to any marked degree in comparisons of " average papillomas per survivor ". When " numbers of papilloma-bearing mice " were compared, the differences were negligible. Because the animals were randomized between groups initially, and because at least one parameter of response to the earlier application of croton oil indicated little difference between groups 2–7, it was felt justifiable to accept the results with regard to malignant tumours as an indication that *prolongation of promoting treatment increases the incidence of malignancy.* The next problem was to ascertain whether the trend in incidence seen between group 2 and group 6 remained as clear-cut after sex-differences and differences in survival had been taken into account. The results were therefore analysed by an actuarial method adapted for the purpose (Pike and Roe, 1963). Mice of each sex were analysed separately and in both cases the tendency for prolongation of croton oil treatment from 10 to 50 weeks to increase malignant tumour incidence remained after differences in survival had been taken into account. In the case of females, but not of males, prolongation of croton oil treatment beyond the 50th week was accompanied by a higher tendency to develop malignant tumours. However, the sizes of the two groups from the 50th week onwards were clearly too small for any confidence to be placed on the apparent differences between them.

In Table III, the incidence of all tumours, other than benign skin tumours and well-circumscribed non-invasive pulmonary adenomas, is recorded. With the possible exception of some of the hepatomas, the malignancy of which is difficult to measure, all the tumours shown in Table III were malignant as judged microscopically. The incidence of malignant tumours other than of the skin showed no obvious relation to treatment.

A somewhat higher incidence of malignant skin tumours was seen in males than in females. On the other hand, malignant lymphoma and reticulum cell sarcoma were seen more than twice as frequently in females.

Between one and five animals in each group had histologically confirmed lung tumours. In addition, several small lung lesions which appeared macroscopically to be adenomas were not taken for section. Taking both histologically confirmed and unconfirmed lesions into account, there appeared to be no relation between their incidence and treatment. Of the total of 23 mice which bore histologically confirmed primary adenomatous tumours of the lung, 12 were males and 11 females. In three mice lung tumours showed macroscopic and microscopic evidence of malignancy. These are recorded in Table III.

Lymph gland metastases from malignant skin tumours were seen in 14 mice and distant metastases in 10 (Table III). The prolongation of croton oil treatment did not appear to increase the proportion of malignant tumours which metastasised. On the other hand, the experiment was not designed to study this point, and the practice of removing malignant skin tumours under anaesthesia and returning animals to the experiment, clearly modified the incidence of metastasis, possibly unequally in the different treatment groups.

In Table IV a comparison is made between (1) the cumulative total of papilloma-bearing mice and the average number of papillomas per survivor 2 weeks after the end of croton oil treatment, and (2) the cumulative totals of malignant tumour-bearing mice and malignant tumours at 90 weeks. All these measures of response to treatment follow the same trend, but it is clear that the ultimate incidence of malignant tumours cannot be more than very roughly predicted from a knowledge of the incidence of benign tumours at the end of croton oil treatment. The fact that 2 malignant and 1 probably malignant tumour arose in group 2 which developed very few papillomas was particularly surprising. It must be emphasised that these analyses of the data do not take into account survival differences between the groups. Nevertheless, we feel justified in concluding that the cumulative total of mice which have borne benign skin tumours up to the time croton oil treatment is stopped (expressed as a percentage of the mice at risk) is at least as valuable in the prediction of the ultimate incidence of malignancy as the average number of papillomas per survivor. In the accompanying paper (Pike and Roe, 1963) the same data are analysed using the former parameter of tumour response, but allowing for intercurrent deaths.

### DISCUSSION

With certain reservations which are discussed below, we feel that the results presented in this paper support the general hypothesis that, in two-stage carcinogenesis, the ultimate incidence of malignancy varies directly with the length of exposure to the " tumour-promoting " stimulus.

The fact that we did not observe a difference in malignant tumour incidence between mice treated for 50 and 77 weeks with croton oil may indicate that beyond a certain point in time of exposure or in the life-span, cessation of exposure to the promoting stimulus has no effect. The relevant data in the present experiment are unfortunately inadequate to throw much light on this problem.

The occurrence of many benign and a few malignant tumours in the two groups treated with croton oil only indicates that, at least for the " S " strain of mice, croton oil is a complete carcinogen. Should we then regard croton oil not as a tumour-promoting agent but simply as a carcinogen ? Should we also modify the wording of the hypothesis expressed in the first paragraph of this section ? Certainly the concept of " pure initiators " and " pure promoters " complementing each other's activities with resulting carcinogenesis, is now a myth. Nevertheless, there still seems to be some value in classifying carcinogens as " of the initiating type " and " of the promoting type ". For mouse skin urethane remains the best example of the former and croton oil a moderately good example of the latter. With these reservations we feel that it is still useful to retain the distinction between " initiators " and " promoters ".

### SUMMARY

1. Following a single application of 300 $\mu$g. 3,4-benzopyrene (BP) in acetone, different groups of mice were painted once weekly with croton oil in acetone for 10, 20, 30, 40, 50, or 77 weeks. Control groups received 300 $\mu$g. BP followed by 50 weekly applications of acetone only, or acetone only initially followed by weekly applications of croton oil for 35 or 77 weeks.

2. The incidence of malignant skin tumours in mice which received BP initially, varied directly with the length of croton oil treatment between 10 and 50 weeks. Continuation of croton oil from 50 to 77 weeks had no marked effect on malignant tumour incidence.

3. A few benign tumours developed in response to 300 $\mu$g. BP followed by acetone only. Many benign and a few malignant tumours arose in mice treated for 35 or 77 weeks with croton oil without BP-pretreatment. The tumours in these groups were at no time as numerous as in the corresponding BP-pretreated groups.

4. The results are examined by an actuarial method in an accompanying paper (Pike and Roe, 1963).

We are most grateful to Mr. E. Woollard and to Miss Susan Bristow for undertaking the histological preparations, and to Mrs. K. Foster for help in preparation of the manuscript.

This investigation has been supported by grants to the Chester Beatty Research Institute (Institute of Cancer Research : Royal Cancer Hospital) from the Medical Research Council, the British Empire Cancer Campaign, the Tobacco Research Council, the Anna Fuller Fund, and the National Cancer Institute of the National Institutes of Health, U.S. Public Health Service.

### REFERENCES

BERENBLUM, I. AND HARAN, N.—(1955) *Brit. J. Cancer*, **9**, 268.

*Idem* AND SHUBIK, P.—(1947*a*) *Ibid.*, **1**, 379.—(1947*b*) *Ibid.*, **1**, 383.—(1949*a*) *Ibid.*, **3**, 109.—(1949*b*) *Ibid.*, **3**, 384.

PIKE, M. C. AND ROE, F. J. C.—(1963) *Ibid.*, **17**, 605,

ROE, F. J. C.—(1956*a*) *Ibid.*, **10**, 61.—(1956*b*) *Ibid.*, **10**, 72.—(1957) *Rep. Brit. Emp. Cancer Campgn*, **35**, 511.—(1959) *Brit. J. Cancer*, **13**, 87.

SALAMAN, M. H. AND ROE, F. J. C.—(1956*a*) *Ibid.*, **10**, 70.—(1956*b*) *Ibid.*, **10**, 79.

SHUBIK, P.—(1950) *Cancer Res.*, **10**, 713.